# *Cornus mas*: From Plant Taxonomy and Distribution Area to Highly Valorization of Phytochemicals by Microencapsulation in Biopolymeric Matrices Containing Probiotics

**DOI:** 10.3390/plants14213298

**Published:** 2025-10-29

**Authors:** Iuliana-Maria Enache, Nicoleta Stănciuc, Aida Mihaela Vasile, Rodica Mihaela Dinică, Eliza Țupu, Camelia Vizireanu

**Affiliations:** 1 Faculty of Horticulture, “Ion Ionescu de la Brad” Iasi University of Life Sciences, 3 Mihail Sadoveanu Alley, 700490 Iasi, Romania; iuliana.enache@iuls.ro; 2 Faculty of Food Science and Engineering, “Dunărea de Jos” University of Galati, 111, Domnească Street, 800201 Galai, Romania; aida.vasile@ugal.ro; 3 Faculty of Science and Environment, “Dunărea de Jos” University of Galati, 111 Domnească Street, 800201 Galati, Romania; rodica.dinica@ugal.ro; 4 Botanical Garden of Natural Sciences Museal Complex “Răsvan Angheluță” Galati, 6A, Regiment 11 Siret Street, 800340 Galati, Romania; elizatupu@yahoo.com

**Keywords:** anthocyanins, microencapsulation, *Cornus mas*, maltodextrin, whey protein isolate, *Lacticaseibacillus casei*

## Abstract

In this study, a comprehensive approach to the taxonomy and the distribution areas of *Cornus mas* (commonly known as cornelian cherry) is presented, considering the superior valorization of bioactive compounds through co-microencapsulation in a unique matrix combination, together with probiotic bacteria. According to the phytochemical profile, the whole plant of cornelian cherry includes 101 chemical compounds, classified as follows: polyphenols, terpenoids, carotenoids, vitamins, carbohydrates, acids, and hydrocarbons. In general, the bioactive compounds are highly sensitive to digestion and external factors, such as oxygen, pH, temperature, etc. In order to improve the bioaccesibility and the storage stability of the polyphenols, a solid–liquid ultrasound assisted method was applied to deliver an anthocyanin-enriched extract, which was microencapsulated together with *Lacticaseibacillus casei (L. casei)* by freeze-drying in a unique combination of whey protein isolate (WPI) and maltodextrin (MD) as wall materials. Two powders were obtained, with and without the probiotic bacteria. The data obtained in this study showed a high encapsulation efficiency (82.16–88.95%) of anthocyanins, whereas for *L. casei*, the microencapsulation efficiency reached 80%. The co-microencapsulated powder showed a viable cell count of 3.80·10^9^ CFU/g dry matter (D.M.). The microencapsulated powders showed a significant amount of total polyphenols (8.30–13.00 mg of gallic acid equivalent per gram D.M.). Furthermore, the in vitro digestibility of the anthocyanins highlighted the protective effect of the microencapsulation matrix in the stomach, whereas a slow release was observed in the simulated intestinal conditions. Furthermore, after 21 days of storage, the lactic acid bacteria viability was high (2.53 × 10^9^ CFU/g dry matter), which confirmed the functionality and the nutraceutical value of the co-microencapsulated powder.

## 1. Introduction

Most of the current research has focused on the beneficial role of plant phytochemicals for human health, including their antioxidant and anti-inflammatory capacities, their antidiabetic and anticancer activities, and their effects on preventing neuronal illnesses or reducing cardiovascular diseases. In this context, numerous studies have highlighted the significant impact of anthocyanins on human health and the advantages provided as naturally occurring, plant-derived, red, violet, and blue pigments [1,2,3]. Additionally, food’s color is one of the most significant organoleptic qualities, indicating both the freshness of foods and its anthocyanin content. The natural anthocyanins have recently received more attention due to the opportunities to replace the synthetic dies in the food industry and as nutraceuticals.

*Cornus mas* L. is one of most important edible forest fruits found in spontaneous crops in Romania (38 areas) [4]. The plant’s bioactive compounds are recognized for their important beneficial effects on asthma, rheumatism, cardiovascular disease, anemia, haemorrhoids, cancer, inflammation, stomach ulcers, colitis, and kidney disorders. Different reports showed that the biologically active components (BACs) found in cornelian cherry fruit are the basis for all of its ethnomedicinal benefits [5,6,7].

However, in order to translate the plant’s bioactivity in foods and nutraceuticals to targeted functions, ongoing concerns were expressed regarding the influence of light, oxygen, storage duration, temperature, pH, etc., on the anthocyanins’ stabilities [8]. Improvements in the functional properties and the protection of the anthocyanins may be achieved by extraction and microencapsulation using different techniques.

The objectives of this work were to establish a comprehensive image of the *Cornus mas* L. potential to be exploited as a source for functional foods and nutraceuticals. Therefore, in the first part of this work, important information is provided regarding the plant composition, its taxonomy, and its cultivation areas in Romania, followed by a proposed strategy to valorize the phytochemicallys enriched extract into freeze-dried powders containing polyphenolic compounds and probiotics microencapsulated in a complex matrix formed by proteins and carbohydrates. Therefore, the wall materials used in this study were chosen based on the different reports in the literature regarding the protective effects on anthocyanins and probiotics. Hence, whey protein isolate (WPI) and maltodextrin (MD) were combined in a certain ratio to obtain a self-stable matrix for the bioactive compounds and the probiotics. WPI is successfully used in the food industry due to its high protein content (over 90%). These high-quality proteins have the ability to supplement the product with nutrients that are beneficial to the human body [9]. Maltodextrin is a polymer with proven efficacy in microencapsulating phenolic compounds due to its appropriate viscosity, affordability, sweet flavor, and practical particle size [10]. *L. casei* belongs to the *Lacticaseibacillus* genus and is used in the food industry for its benefits to human health. The probiotic effects of *Lacticaseibacillus* include improving lactose digestion, reducing its intolerance symptoms [11]. The phytochemical extraction was performed by a solid–liquid ultrasound assisted extraction method, using ethanol as the solvent, followed by microencapsulation in two variants, described as follows: microencapsulation of the extract in a biopolymeric matrix containing WPI and MD (variant 1) and a co-microencapsulated variant containing WPI, MD, and *L. casei* 431^®^ (LC) (variant 2) by freeze-drying. The powders were tested for total polyphenolic content, cell viability, in vitro digestion, and color and storage stability.

The main aim of this approach is to give a holistic view of the cornelian cherry industrial potential, from the plant to foods and nutraceuticals.

## 2. Results

### 2.1. Taxonomic Classification and Worldwide Distribution of Cornus mas

Cornelian cherry is a woodland fruit belonging to the kingdom *Plantae*, the infra-kingdom *Viridiplantae*, the super-kingdom *Streptophyta*, the sub-kingdom *Embryophyta*, the class *Magnoli*-opsyda, the super-order *Asteranae*, the order *Cornales*, the family *Cornaceae*, the genus *Cornus* L., and the species *Cornus mas* [12,13]. Over time, 28 horticultural forms and variants of *Cornus mas* L. have been discovered. They are as follows: *Cornus mas* f. *alba (Weston)* Rehder; *Cornus mas* var. *albocarpa* (C.K. Schneid.) Bean; *Cornus mas* f. *andrzejowskii* Wierdak; *Cornus mas* f. *aurea* C.K. Schneid; *Cornus mas* f./var. *aurea-elegantissima* (T. Moore), Schelle, Carriere; *Cornus mas* f. *conica* Jovan; *Cornus mas* f. *elegantissima* G. Nicholson; *Cornus erythrocarpa* St.-L; *Cornus mas* f./var. *flava infra* subsp. Publ (Weston) Rehder, Steud; *Cornus homerica* Bubani; *Cornus mas* f. *luteocarpa* (Wangerin), C.K. Schneid; *Cornus mas* f./var. *macrocarpa* (Dippel) Schelle; *Cornus mas* f. *microcarpa* Sanadze; *Cornus mas* f./var. *nana* (Carriere) C.K. Schneid; *Cornus mas* var. *oblongifolia* Jovan; *Cornus mas* f. *oxycarpa* Jovan; *Cornus mas* f. *polonica* Wierdak; *Cornus mas* f. *pyriformis* Sanadze; *Cornus mas* var. *sphaerocarpa* Cretz; *Cornus mas* f./var. *variegate* (Loudon) G. Nicholson; *Cornus mas* var. *xanthocarpa* Bean; *Cornus mascula* L.; *Cornus mascula* var. *aurea-elegantissima* T. Moore; *Cornus nudiflora* Dumort; *Cornus praecox* Stokes; *Cornus vernalis* Salisb; *Eukrania mascula* (L.) Merr.; *Macrocrpium mas* (L.) Nakai [14].

In terms of geographical distribution, *Cornus mas* L. is cultivated in Albania, Armenia, Austria, Azerbaijan, Belgium, Bosnia and Herzegovina, Bulgaria, Czech Republic, Croatia, France, Germany, Georgia, Greece, Northern Iraq, Iran, Italy, Lebanon, Luxembourg, the Balkan Peninsula, Poland, Romania, Russia, Serbia and Montenegro, Hungary, Slovakia, Slovenia, Switzerland, Syria, Ukraine, and Turkey [15], but is also used in countries such as China, Kosovo, and the United States of America [16,17]. The genus *Cornus* includes shrub species widely distributed in southern and central Europe, southwest Asia, America (north, east, south, west, and central), and eastern Africa [18]. Two species of this genus are endemic to South America and one species to tropical Africa [16,17]. Regarding the worldwide area cultivation of cornelian cherry (in ha), Georgia occupies 0.3% of the total fruit orchards (135 ha) [19], while Turkey has 1.6 million cornelian cherry trees producing 17,000 tons of fruit annually. In addition, in this country, 97% of cornelian cherry fruit production is harvested from wild flora [20]. Meanwhile, in Romania, *Cornus mas* (CM) is found in 38 areas and can be considered one of the most important forest fruits in our country. It is well known that the altitude, temperature, average annual precipitation, relief, rock type, soil type, phytocenosis structure, and floristic composition of the habitat can influence *C. mas* distribution in our country [4,8]. A native from the Caucasus [21,22], CM is one of four species that are harvested for their fruits (*C. mas*, *C. officinalis*, *C. controversa*, and *C. kousa*) and not only for ornamental purposes, as are the other species of *Cornus* [23,24]. Regarding the number of species in the *Cornaceae* family, different reports are available, such as the following: 40 species [25], 55 species [26,27], 60 species [28], and 65 species (15 red-line species and 50 blue-line species) [5,18,24].

**Figure 1:**
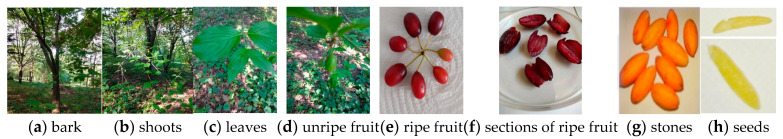
*Cornus mas* plant (original photos).

In Figure 1, some suggestive segments of the CM plant (bark, shoots, leaves, fruits, stone, and seeds) are shown.

A study by Dinda et al. [5] provides an overview of the phytochemical profile of the whole plant of *Cornus mas*. Briefly, the cornelian cherry contains 101 phytochemical compounds, classified into anthocyanins (10), catechins (4), flavonoids (20), phenolic acids and tannins (11), monoterpenoids (9), triterpenoids (1), iridoids (5), carotenoids (10), vitamins (4), carbohydrates (3), organic acids (8), fatty acids (6), and hydrocarbons (10) (Table 1).

In addition, Table 2 shows the composition of proteins, amino acids, fatty acids, ma- croelements, and microelements found in cornelian cherry fruit pulp.

### 2.2. Phytochemical Characterization of the Extract from Cornelian Cherry

The data obtained in this study shows that cornelian cherry contains significant levels of polyphenols (17.41 ± 0.67 mg GAE/g D.M.), flavonoids (5.64 ± 0.28 mg EC/g D.M.), and total anthocyanin content (18.27 ± 0.91 mg C3G/g D.M.), as well as a relevant antioxidant activity (185.15 ± 1.15 mMol TE/g D.M.).

The polyphenolic content was analyzed in comparison with the data available in the literature (Table 3).

As it can be observed, the efficiency of the extraction of the total anthocyanins, flavonoids, and polyphenols is highly dependent on the extraction method and the solvent used. The cornelian cherry extract obtained in our study showed a higher global polyphenolic content, which impacted the antioxidant activity.

### 2.3. Co-Microencapsulation Efficiency and Phytochemical Profile of the Powders

From Table 4, it can be observed that the variant with *L. casei* showed significantly (*p* < 0.05) higher values compared to the sample without lactic acid bacteria for encapsulation efficiency and phytochemical profile and comparable values for antioxidant activity.

The anthocyanin microencapsulation efficiency was approximatively 7% higher for the WPI-MD-LC variant, the powder showing an approximatively 16% higher content in anthocyanins and polyphenols and 9% in flavonoids. The higher content of global polyphenolic content influenced the antioxidant activity. A satisfactory co-microencapsulation of 80% was obtained for the lactic acid bacteria, which accounted for 3.80 × 10^9^ CFU/g D.M. The higher encapsulation efficiency may be explained by the enhanced potential interactions between the whey proteins and the polyphenols due to hydrogen bonding and hydrophobic associations, which may enhance the pigment and the probiotic stability within the biopolymeric matrix.

A recent study used WPI, inulin, chitosan, and *Lactobacillus casei* as wall materials to protect the cornelian cherry anthocyanins. The data obtained for the phytochemical profile of the powders demonstrated that the polyphenolic content was lower than in the present study (2.66 ± 0.08 mg GAE/g D.M.). Moreover, the antioxidant activity is significantly higher in this study (15.89 ± 0.19 mM Trolox/g D.M.) compared to the reference above mentioned [36].

Vasile et al. [37] microencapsulated black bean anthocyanins with *L. casei*, chitosan, and inulin from chicory. The data obtained showed that the anthocyanins had an encapsulation effectiveness of 99.33 ± 0.13%, whereas the lactic bacteria had a value of 77.42 ± 1.34%. Furthermore, Colin-Cruz et al. [38] suggested that wall materials have a significant influence on the encapsulation efficiency rate. These authors used spray-drying microencapsulation of blackberry (*Rubus fruticosus*) bioactive compounds from juice in whey protein concentrate, acacia gum, and maltodextrin and reported a microencapsulation efficiency of anthocyanin ranging from 71.90 to 93.00%.

### 2.4. In Vitro Digestion of Anthocyanins

Due to their structural particularities and pH-dependent sensitivity, the bioavailability of anthocyanin was reported to be in the range of 0.26–1.8% [39]. In order to increase the bioaccesibility of anthocyanin, the cornelian cherry extract was microencapsulated in a biopolymeric matrix containing WPI and MD in a ratio of 1:1. Both of the freeze-dried powders exhibit a good stability of the anthocyanin content during simulated stomach digestion, proving that wall matrices can protect the anthocyanins from cornelian cherry fruits. The good stability of anthocyanin in SGJ may be explained by a glycosyl moiety bonded to anthocyanin, thus affecting the anthocyanin metabolism. Therefore, it can be assumed that the flavylium cation form of anthocyanin is the most abundant in acidic environments such as the stomach, as explained by McGhie & Walton [40].

On the other hand, during intestinal digestion, a controlled release can be observed from Table 5, with an increase of anthocyanin content of 22–25% after 120 min when compared with the beginning of digestion. This may be explained by the impact of the increased pH in the SIJ, leading to the formation of the blue quinonoidal structure generated by the loss of a proton [40]. The increase in anthocyanin content was explained by Fang [41] and is related to the higher degradation rate of anthocyanin at pH values higher than 7.0. In the neutral to alkaline range of the pH scale, the anthocyanins are metabolized to anthocyanidin by the removal of sugars in the intestine, followed by degradation to smaller compounds such as protocatechuic acid and ferulic acid. The results obtained confirm that both of the microencapsulated powders provided a satisfactory protection of anthocyanin in the gastric environment and a slow release in the small intestine, thus improving the bioaccesibility.

The in vitro experimental results demonstrate the beneficial impact of the wall materials to preserve the bioactive compounds, particularly the anthocyanins from CM fruits. The information gathered in this study confirms that the wall materials used can successfully protect the cornelian cherry fruit’s anthocyanins against internal and external factors.

### 2.5. Colorimetric Analysis

Color remains one of the important parameters that influences consumers’ acceptance of products. The colorimetric study of the microencapsulated powders revealed a red hue because of the positive value for the a* parameter, which can be associated with the anthocyanin content. On the other hand, a significant quantity of carotenoid compounds (such as β-carotene) can be linked to a positive value for the b* parameter. The light shade of the wall materials employed—WPI, MD, and lactic acid bacteria—may all be related to the luminosity of both the samples (positive L* values) (Table 6).

The L* parameter values suggest the brightness of the powders due to the biopolymeric matrices. That may be connected to the choice of less intense wall material tones. Furthermore, the addition of lactic acid bacteria powder can be linked to the pink–red shades. Consequently, when compared, the value obtained for the a* parameter of the WPI-MD-LC powder (25.01 ± 0.65) is consistent with the results for the slightly higher content of anthocyanins (12.86 ± 0.86 mg C3G/g D.M.). According to the color wheel, which is the base of the color parameters calculation, the Hue values place both the samples into the red–yellow shades.

### 2.6. Viability of Lactic Acid Bacteria During Storage

During the storage period of WPI-MD-LC variant, a good stability of the viable cells was observed, with a decrease from 3.80 × 10^9^ CFU/g to 3.26 × 10^9^ CFU/g after 7 days, t6 2.89 × 10^9^ CFU/g D.M. after 14 days, and to 2.53 × 10^9^ CFU/g D.M. after 21 days. Therefore, a 66.5% viability of the probiotic was estimated during the 21 days of storage at 4 °C. The decrease in viable cells was described by a first order kinetic model, allowing us to estimate the degradation rate as 52.08 ± 4.35 days with a half time of 0.013 ± 0.0009 days. The remarkable stability of the viable cells over 21 days at 4 °C may be attributed to the prebiotic activity of both the MD and the polyphenols. For example, Oluwatosinr et al. [42] reported values for *L. plantarum* of 13% after 12 weeks of storage under refrigeration when encapsulating the probiotic with MD. Even though the prebiotic activities of polyphenols are not fully scientifically demonstrated in the literature, Medeiros Alves-Santos et al. [43] highlighted that, in animal studies, the consumption of polyphenols, especially catechins, anthocyanins, and proanthocyanidins increased the abundance of *Lactobacillus, Bifidobacterium, Akkermansia, Roseburia*, and *Faecalibacterium* spp.

A similar decreasing behavior in cell viability was reported by Milea et al. [44], who microencapsulated flavonoids from yellow onion skin with WPI, inulin, MD (2:1:1), and *L. casei*. After 21 days of storage at 4 °C in the dark, the viability of the probiotic bacteria was lower by 7.51 log CFU/g D.M. Moreover, Enache et al. [45] have demonstrated the protective effect of the wall materials (WPI, inulin, and *L. casei* or WPI, casein, and *Lactobacillus casei*) when encapsulating cornelian cherry fruit anthocyanins, even after 90 days of storage in dark conditions at 4 °C (9.58 log CFU/g D.M.). However, when considering the viable cell counts, in order to have a powder with probiotic properties, it is essential to maintain a minimum 6 log of viable cells/g by the end of the storage time.

### 2.7. Storage Stability of the Bioactive Compounds in the Encapsulated Powders

The microencapsulated powders were evaluated for antioxidant activity and polyphenol, anthocyanin, and flavonoid content every 7 days throughout a 28-day period of storage in dark conditions at 4 °C. The data are shown in Table 7.

According to the data shown in Table 7, a significant decrease (*p* < 0.05) was observed in the microencapsulated cornelian cherry in WPI and MD without the addition of a probiotic in total polyphenols (18%), which affected the antioxidant activity (a decrease of about 46% for the powder without the probiotic). No significant differences were observed in the flavonoid and anthocyanin content. For the co-microencapsulated powder, a good storage stability was observed in terms of polyphenols, flavonoids, and anthocyanin, whereas the antioxidant activity significantly decreased after 21 days of storage (45%). It can be suggested that in the co-microencapsulated variant, some other compounds may be responsible for the decrease in the antioxidant activity, which can be correlated with the metabolic activity of the probiotics.

## 3. Materials and Methods

### 3.1. Materials

#### 3.1.1. Chemicals and Reagents

The reagents used in this study were the following: acetic acid, aluminum chloride, hydrochloric acid, 2,2-diphenyl-1-picrylhydrazyl (DPPH), 6-hydroxy-2,5,7,8-tetramethylchromane-2-carboxylic acid (Trolox), ethanol, Folin–Ciocâlteu reagent, gallic acid, maltodextrin (dextrose equivalent (DE) of 16.5–19.5), methanol, pancreatin (Kreon), pepsin from gastric porcine, potassium acetate, and Trizma hydrochloride. The reagents were purchased from Sigma Aldrich (Taufkirchen, Germany). Sodium bicarbonate was purchased from Honeywell, Fluka (Selze, Germany), and whey protein isolate 894 (WPI) was purchased from Fonterra (Clandeboye, New Zealand). Lactic acid bacteria *Lactobacillus casei* 431^®^ strain was purchased from Chr. Hansen (Hoersholm, Denmark). *L. casei* cell viability was performed using de Man, Rogosa, and Sharpe agar (MRS agar) purchased from Merck (Darmstadt, Germany).

#### 3.1.2. Fresh Fruit Processing

Fresh fruits of cornelian cherry were collected from Slivna village (Galati, Romania) in September 2019 and then identified by PhD botanist biologist Țupu E. The next step was to wash with distilled water and dry with a paper towel in order to remove all impurities. Finally, the stone seeds were manually removed. The resulting samples were immediately processed by freeze-drying.

### 3.2. Methods

#### 3.2.1. Phytochemical Extractions

To prevent the breakdown of bioactive components, the fruits were freeze-dried (CHRIST Alpha 1-4 LD plus, Osterode am Harz, Germany) at −42 °C under 10 Pa pressure for 48 h. The obtained material was collected and packed. After grinding, 100 g of freeze-dried fruit was mixed with 400 mL of 70% ethanol and extracted for 30 min at 40 °C, 100 W, and 40 KHz (MRC Scientific Instrument 20 L, 50 kHz, Holon, Israel). The extraction parameters (temperature, duration, ultrasonic power, solvent–solid ratio) were chosen in accordance with similar studies already published [32,44]. The hydroalcoholic extract was then centrifuged (Universal 320R, Tuttlingen, Germany) at 5000 rpm for 30 min at 4 °C. The extraction was performed twice. Concentration under vacuum at 40 °C was used to remove the solvents (AVC 2-18, Christ, UK). To prepare the microencapsulated material, the concentrated extract was weighed, dissolved in 20 mL of ultrapure water (UW), filtered, and stored in the refrigerator at 4 °C in dark conditions.

#### 3.2.2. Microencapsulation of the Anthocyanins

The concentrated extract of the cornelian cherry fruits was combined with the chosen wall materials to protect the anthocyanins by freeze-drying microencapsulation. For this purpose, 2 g of concentrated extract of *Cornus mas* fruit was rehydrated with 20 mL of UW for each experimental variant. One variant involved the mixture of 20 mL of extract in UW in 100 mL of MD and WPI (1:1 ratio). The same protocol was applied for co-microencapsulation, with an additional step of inoculation with 1 g of *L. casei*. The pH of both samples was settled at 4.6 to allow for good stability of probiotics and anthocyanins. The mixtures were constantly homogenized for 4 h at 650 rpm with a magnetic stirrer (IKA, Deutschland, Germany). After that, both samples were stored at −4 °C for 24 h to ensure the complete hydration of the ingredients, followed by freeze-drying (CHRIST Alpha 1-4 LD plus, Osterode am Harz, Germany) for 48 h at −42 °C and 10 Pa pressure. The resulting powders were packed in sterile bags and stored at 4 °C in a dark environment for further use.

#### 3.2.3. Phytochemical Profile of the Extract and Freeze-Dried Powders

Total polyphenol content (TPC), total anthocyanin content (TAC), total flavonoid content (TFC), and total antioxidant activity (TAA) were quantified to determine the phytochemical profile of cornelian cherry extract and microencapsulated powders.

TPC was carried out using the Folin–Ciocalteu method according to the protocol: 15.8 mL UW, 0.2 mL of sample previously diluted 1:10, and 1 mL of the Folin–Ciocalteu reagent were thoroughly mixed and shaken. After 10 min, three milliliters of sodium carbonate (3%) were added. After 60 min in complete darkness, the samples’ absorbance was measured at 765 nm using a UV-Vis Jenway spectrophotometer (OSA, UK). The results were expressed as mg gallic acid/g D.M. based on a calibration curve [45].

By mixing 0.250 mL of diluted liquid sample (1:10) with 1.25 mL UW and 0.075 microliters of sodium nitrate (5%), total flavonoid compounds were measured. After five minutes, 0.15 mL of aluminum chloride (10%) was added. After six minutes, the sample was mixed with 0.77 mL UW and 0.5 mL sodium hydroxide (1 M). The same spectrophotometer was used to instantly read the absorbance at 510 nm, and the results were expressed as mg equivalent catechins/g D.M. [45].

For total anthocyanin content (TAC), as well as for surface anthocyanin content (SAC), the same spectrophotometer was used to quickly read the absorbance at 520 nm and 700 nm at two pH values. Using two buffers (buffer 1: potassium chloride at pH 1 and buffer 2: sodium acetate at pH 4.5), TAC was calculated according to Equations (1) and (2) [46]:

A = (pH_1_ − pH_4_._5_)_520_ − (pH_1_ − pH_4_._5_)_700_ (1)

(2) $$TAC\;\left(Cyanidin\;3-glucoside\;equivalents,\frac{mg}{g\;DM}\right)=\frac{A\times MW\times DF\times 10^{3}}{\varepsilon \times l}\times \frac{V}{W}$$

where

A—the differences in absorbance, calculated using Equation (1);

MW—molecular weight of cyanidin-3-glucoside (449.2 g/mol);

DF—dilution factor;

ε = molar extinction coefficient for cyanidin-3-glucoside (26,900 L/(mol·cm);

l = width of the cuvette in cm;

W = sample weight (mg);

V = volume in mL;

10^3^ = conversion factor from g to mg.

The total antioxidant activity of the samples had been performed using the DDPH method, earlier described by Oancea et al. [46]. Briefly, 100 microliters of sample were mixed with 3900 microliters of DPPH solution. After 90 min in dark conditions, the absorbance was measured with a spectrophotometer at 515 nm. The data were finally presented as mMol Trolox equivalent/g D.M., based on the calibration curve [46].

#### 3.2.4. Viability of Lactic Acid Bacteria

The pour plate method, MRS-agar (De Man, Rogosa, and Sharpe medium at pH 5.7), and sterile saline (0.9 g NaCl, *w*/*v*) were used to measure the survival of lactic acid bacteria with probiotic potential. The ISO 8261 [47] method was applied to determine the quantity of live *L. casei* 431^®^ cells. This involved counting the number of live cells, which was done on MRS-agar after 48 h at 37 °C in an aerobic environment. Colony forming units (CFU/g D.M.) were used to express the viable cell count. The initial viable cell count was 10^11^/g D.M.

#### 3.2.5. Microencapsulation Efficiency of the Powders (%)

The quantification of total anthocyanin content (TAC) and surface anthocyanin content (SAC) in accordance with Equation (1) was used to determine the anthocyanin encapsulation efficiency (AAE) of the two microencapsulated powders.

(3) $$AAE\left(\%\right)=\frac{TAC-SAC}{TAC}\times 100$$

For TAC protocol, methanol, acetic acid, and ultrapure water were used in a ratio of 5:0.8:4.2 (*v*/*v*/*v*), whereas ethanol and methanol were mixed in an identical ratio (*v*/*v*) to measure SAC concentration. About 200 mg of powder was mixed with 5 mL of the corresponding solvent. In order to measure the total anthocyanin content, the samples were submitted to ultrasound treatment for 15 min at 40 °C, 100 W, and 40 KHz (MRC Scientific Instrument 20 L, 50 kHz, Holon, Israel) to allow the breakup of the microparticles and the release of the anthocyanin.

Following the procedure outlined by Colin-Cruz et al. [38], the co-microencapsulation efficiency (MEE) of *L. casei* was quantified. The number of viable cells in the powder (N) and the number of viable cells in the mixture just before freeze-drying (N0), both expressed as CFU/g D.M., were used to calculate the efficiency of co-microencapsulation (%) using Equation (4) [43].

(4) $$MEE\left(\%\right)=\frac{N}{N0}\times 100$$

#### 3.2.6. In Vitro Digestion of Anthocyanins

A static model was used to investigate the in vitro digestibility of anthocyanins using simulated gastric juice (SGJ) and simulated intestinal juice (SIJ). Briefly, in a hermetically sealed plastic flask, 500 mg of sample was mixed with 10 mL Trizma hydrochloride (0.05 M, pH = 7) and 20 mL SGJ (20 mL HCl 0.1 N and 20 g pepsin, pH = 2), followed by rapid agitation until complete homogenization. After that, each microencapsulated powder in SGJ was maintained at 37 °C in an orbital shaker for 120 min. Every 30 min, 2 mL of sample was drawn, centrifuged (at 14,000 rpm for 10 min at 4 °C), and the anthocyanin concentration was calculated in accordance with the above-mentioned methodology. After 2 h, 5 mL of SGJ was mixed with 20 mL of SIJ (20 mg pancreatin and 10 mg pepsin, pH = 7.8), and the same protocol was used to sample and measure the anthocyanin content every 30 min [46].

#### 3.2.7. Colorimetric Analysis

A CR Konica Minolta (Japan) food colorimeter and a CIE L*a*b* system, a scientific tool, were used to analyze the color properties of the two powders. The scientific instrument’s display shows three variables: L* (which represents the brightness of the examined samples) and two color characteristics, a* and b*. The parameter a* identifies shades of green (negative values) or red (positive values). The parameter b* quantifies shades of blue (negative values) or yellow (positive values), while for L*, factor values range between 0 (for black) and 100 (for white). According to the procedure outlined previously by Browning et al. [48] and Enache et al. [45], two additional coordinates (C*, h*) could be calculated.

#### 3.2.8. Storage Stability

Using the methodologies outlined above, the following tests were carried out to quantify the storage stability over time: total anthocyanin content, total polyphenol content, total flavonoid content, and total antioxidant capacity. The two experimental variants of storage stability were examined by repeating the test procedure after 7, 14, 21, and 28 days (codified as T7, T14, T21, T28) in dark conditions at 4 °C and 50–60% relative humidity.

### 3.3. Statistical Analysis

Using the Microsoft Excel Package Tool, each analysis for this study was carried out in triplicate, and the data were presented as means and standard deviations. Based on the Tukey test method and the 95% confidence interval with *p* < 0.05, the statistical difference between the samples was determined.

## 4. Conclusions

The data presented in this study highlighted the remarkable polyphenolic content of cornelian cherry extract, obtained by ethanol–water ultrasound assisted extraction. The extract was micro- and co-microencapsulated using a combination of WPI and MD, allowing us to obtain two powders with a high content in total anthocyanin, polyphenols, and flavonoids and a strong antioxidant activity. However, when the extract was co-microencapsulated with probiotics, a higher anthocyanin encapsulation efficiency was found, together with a satisfactory level of viable cells trapped in the biopolymeric matrices. Additionally, the microbial load reached 3.80 × 10^9^ CFU/g D.M. after freeze-drying, with a good stability during storage, suggesting a potential nutraceutical and functional effect. Additionally, the protective effect of the matrix used was demonstrated by the in vitro digestibility of the anthocyanins. Accordingly, the increased bioaccesibility of the anthocyanins in the gastrointestinal simulated media was demonstrated by the good stability in gastric conditions and a slow release in the intestinal environment. Moreover, the colorimetric analysis revealed red–yellow shades for both of the experimental variants tested due to the presence of anthocyanins (positive value for the a* parameter related to the red color) and carotenoids (positive value for the b* parameter associated with yellow shades).

Further studies will be developed in order to highlight the polyphenolic fingerprint of the extract and powders in order to have a complete picture of the potential metabolic activity of the co-microencapsulated powder on the polyphenolic profile.

## Figures and Tables

**Table 1:** Phytochemical profile of *Cornus mas* [5,29].

No.	Chemical Category	Phytochemical Compounds	Plant Organ
1.	Polyphenolic structure compounds		
1.1	Anthocyanins	cyanidin 3-0 galactoside (1), cyanidin-3-O-glucoside (2), cyanidin 3-O-rutinoside (3), cyanidin 3-O-robinobioside (4), delphinidin-3-O-galactoside (5), pelargonidin3-O-galactoside (6), pelargonidin-3-O-glucoside (7), pelargonidin3-O-rutinoziside (8), peonidin3-O-glucoside (9), pelargonidin 3-O robinobiozid (pelargonidin 3-*O*-rhamnosylgalactoside) (10)	fruits
1.2.	Catechins	(+) catechin (11), (-) epicatechin (12), procyanidin B1 (13), procyanidin B2 (14)	fruits, leaves
1.3.	Flavonoids	kampferol-3-O-galactoside (15), kaempferol 3-O glucoside (16), kaempferol 3-O-glucuronide (17), isorhamnetin 7-O-ramnozide (18), myricetin (19), 3-O-methyl ester naringenin (20), quercetin (21), quercetin 3-O-xyloside (22), quercetin 3-O-ramnoside (23), quercetin 3-O-glucoside (24), quercetin 3-O-galactoside (25), quercetin 3-0-, trans-aromadendrin (26), quercetin 3-O-robinobioside (27), quercetin 3-O-rutinoside (rutin) (28), quercetin 3-O-glucuronide (29), quercetin 3-O-galactozil 7-O-ramnoside (30), trans aromadendrin (31), 7-acetoxy-5,2′,4′,6′-β-pentahydroxy-3-methoxychalcone (32), 7-O-aromadendrin glucoside (33), 7,3′dihydroxy-5,4′dimethoxyflufonone (34)	flowers, leaves, fruits
1.4.	Phenolic acids and tannins	quinic acid (35), chlorogenic acid (36), ellagic acid (37), ferulic acid (38), gallic acid (39), salicylic acid (40), shikimic acid (41), vanillic acid (42), p-coumaric acid (43), 3-O-cafenoylquinic acid (44), 5-O cafenoyl quinic acid (45)	flowers, fruits
2.	Terpenoids		
2.1.	Monoterpenoids	a-terpeneol (46), borneol (47), camphor (48), carvone (49), carvacrol (50), limonen (51), verbenone (52), β-tujon (53), 1,8-cineole (54)	flowers
2.2.	Triterpenoids	ursolic acid (55)	leaves
2.3.	Iridoids	loganic acid (56), cornusid (57), loganin (58), sverosid (59), secologanin (60)	fruits
3.	Carotenoids	(all-E)-neoxanthin (61), β-carotene-5,6-monooxide (62), β-carotene (63), β-cryptoxanthin (64), lutein (65), lutein-5,6-epoxide (66), luteoxanthin (67), (9Z,9′Z)-lutein (68), (9′Z)-neoxanthine (69), (13Z,13′Z)-lutein (70)	fruits
4.	Vitamins	ascorbic acid (71), α-tocopherol (72), biotin (73), riboflavine (74)	fruits
5.	Carbohydrates	glucose (75), sucrose (76), fructose (77)	fruits
6.	Acids		
6.1.	Fatty acids	linoleic acid (78), oleic acid (79), a-linolenic acid (80), palmitoleic acid (81), palmitic acid (82), 2,4-heptadienoic acid (83)	fruits, leaves
6.2.	Organic acids	citric acid (84), fumaric acid (85), isocitric acid (86), maleic acid (87), malonic acid (88), oxalic acid (89), succinic acid (90), tartaric acid (91)	fruits, leaves
7.	Hydrocarbons	decane (92), undecane (93), dodecane (94), pentadecane (95), hexadecane (96), heptadecane (97), heneicosane (98), tricosane (99), pentacosane (100), heptacosane (101)	flowers

**Table 2:** Protein, amino acid, fatty acid, and macro- and microelement content of *Cornus mas* [30].

Crt. No.	Identified Compound	Results	Unit of Measurement
**A.**	**Proteins and amino acids**		
1.	Protein	3.40	g/kg
2.	Aspartic acid	0.30	g/kg
3.	Glutamic acid	0.30	g/kg
4.	Arginine	0.30	g/kg
5.	Lysine	0.30	g/kg
6.	Glycine	0.20	g/kg
7.	Phenylalanine	0.20	g/kg
8.	Leucine	0.20	g/kg
9.	Isoleucine	0.20	g/kg
10.	Valine	0.20	g/kg
11.	Alanine	0.10	g/kg
12.	Histidine	0.10	g/kg
13.	Proline	0.10	g/kg
14.	Serine	0.10	g/kg
15.	Threonine	0.10	g/kg
16.	Tyrosine	0.10	g/kg
**B.**	**Fatty acids**		
17.	Linoleic acid	76.70	100 g D.M.
18.	Palmitic acid	39.60	100 g D.M.
19.	Linolenic acid	27.50	100 g D.M.
20.	Oleic acid	18.30	100 g D.M.
21.	Pentadecanoic acid	5.10	100 g D.M.
22.	Stearic acid	4.40	100 g D.M.
23.	Vaccenic acid	3.70	100 g D.M.
24.	Myristic acid	3.50	100 g D.M.
25.	Lauric acid	1.80	100 g D.M.
26.	Palmitoleic acid	1.40	100 g D.M.
**C.**	**Macro- and microelements**		
27.	Potassium	7.04–20.32	g/kg
28.	Nitrogen	3.15–6.44	g/kg
29.	Calcium	0.47–2.59	g/kg
30.	Phosphorus	0.42–1.36	g/kg
31.	Sulfur	0.39–1.64	g/kg
32.	Magnesium	0.29–1.89	g/kg
33.	Iron	6.00–171.00	mg/kg
34.	Pigments	3.43–37.25	mg/kg
35.	Zinc	3.30–41.00	mg/kg
36.	Copper	1.20–8.10	mg/kg
**D.**	**Dry matter**	58.50–83.00	%

**Table 3:** Phytochemical profiles of different cornelian cherry extracts.

Identified Compound	Extraction Method	Solvent Extraction	Results	Unit of Measurement	Reference
Total anthocyanin content	ultrasound	Ethanol 60%, ethanol 80%	189.70–261.84	mg C3G/100 g D.M	[31]
	ultrasound	Hot water	1.23 ± 0.10	mg C3G/g DW	[32]
Total polyphenolic content	ultrasound	Ethanol 60%, ethanol 80%	1700.64–4854.61	mg GAE/100 g D.M.	[31]
	ultrasound	20 mL methanol + 2% HCl	163.69 ± 0.04 (S1) to 359.28 ± 9.57 (H2)	mg GAE/100 g FW ^1^	[33]
Total flavonoid content	Soxhlet method	Water Petroleum ether Methanol Acetone Ethyl actate	3.53 ± 0.39 6.91 ± 0.09 7.18 ± 0.10 8.05 ± 0.76 41.49 ± 0.57	mg Rutin/g extract	[34]
	ultrasounds	20 mL methanol + 2% HCl	12.14 ± 0.01 (S1) to 64.48 ± 0.81 (H3)	mg QE ^2^/100 g FW	[33]
Total antioxidant activity	maceration/stirring	100 mL acetone	677.88 ± 19.25 (ABTS ^3^ assay) 628.75 ± 17.41 (FRAP ^4^ assay)	µmol Trolox equivalents/100 g FW	[35]
	ultrasound	20 mL methanol + 2% HCl	1.24 ± 0.01 (H1) to 2.71 ± 0.05 (H3)	mmolTrolox/100 g FW	[33]

^1^ FW—fresh weight; ^2^ QE—Quercetin Equivalent; ^3^ ABTS—2,2′-azino-bis3-ethylbenzothiazoline-6-sulfonic acid; ^4^ FRAP—Ferric Reducing Antioxidant Power Assay.

**Table 4:** Microencapsulation efficiency, phytochemical content, and *L. casei* content of the powders.

Analyzed Parameter	WPI-MD	WPI-MD-LC
Anthocyanin microencapsulation efficiency (%)	82.16 ± 0.78 ^b^	88.95 ± 0.42 ^a^
*Lacticaseibacillus casei* 431^®^ microencapsulation efficiency (%)	N.A.	80
Total anthocyanin content (mg C3G/g D.M.)	11.16 ± 0.53 ^b^	12.86 ± 0.86 ^a^
Total polyphenolic content (mg GAE/g D.M.)	11.17 ± 0.11 ^b^	12.95 ± 0.38 ^a^
Total flavonoid content (mg CE/g D.M.)	4.97 ± 0.17 ^b^	5.43 ± 0.09 ^a^
Total antioxidant activity (mMol TE/g D.M.)	62.53 ± 1.17 ^a^	63.40 ± 0.88 ^a^
*Lactobacillus casei* 431^®^ (CFU/g D.M.)	N.A.	3.80 × 10^9^

N.A.—not applicable; WPI-MD—microencapsulated extract from cornelian cherry fruits with whey protein isolates and maltodextrin; WPI-MD-LC—microencapsulated extract from cornelian cherry fruits with whey protein isolates, maltodextrin, and *L. casei*. For each phytochemical analysis, values for the studied powders that share different lowercase letters (^a^, ^b^) are statistically different at *p* < 0.05, based on the Tukey test.

**Table 5:** In vitro digestibility of the anthocyanins.

Time (min)	WPI-MD	WPI-MD-LC
**Simulated gastric juice (SGJ)**		
T0 (mg C3G/g D.M.)	7.96 ± 0.48 ^a,B^	9.03 ± 0.37 ^b,A^
T30 (mg C3G/g D.M.)	8.25 ± 0.74 ^a,A^	9.14 ± 0.89 ^b,A^
T60 (mg C3G/g D.M.)	8.42 ± 0.53 ^a,A^	9.62 ± 0.66 ^ab,A^
T90 (mg C3G/g D.M.)	8.46 ± 0.37 ^a,B^	10.87 ± 0.43 ^a,A^
T120 (mg C3G/g D.M.)	8.74 ± 0.47 ^a,B^	10.90 ± 0.21 ^a,A^
**Simulated intestinal juice (SIJ)**		
T0 (mg C3G/g D.M.)	12.41 ± 0.18 ^c,A^	12.86 ± 0.89 ^c,A^
T30 (mg C3G/g D.M.)	13.22 ± 0.56 ^bc,A^	12.98 ± 0.17 ^c,A^
T60 (mg C3G/g D.M.)	13.69 ± 0.56 ^abc,A^	13.73 ± 0.61 ^bc,A^
T90 (mg C3G/g D.M.)	14.51 ± 0.63 ^ab,A^	15.63 ± 0.87 ^ab,A^
T120 (mg C3G/g D.M.)	15.09 ± 0.96 ^a,A^	16.15 ± 0.78 ^a,A^

WPI-MD—microencapsulated extract from cornelian cherry fruits with whey protein isolates and maltodextrin; WPI-MD-LC—co-microencapsulated extract from cornelian cherry fruits with whey protein isolates, maltodextrin, and *L. casei*. Different lowercase letters (^a^, ^b^, ^c^) indicate that the values in the same collum for SGJ and SIJ are statistically different (*p* < 0.05), while different upercase letters (^A^, ^B^) mean that the digestion time is the same for SGJ and SIJ but correspond to different analyzed powders, and they are statistically different at *p* < 0.05, based on the Tukey test.

**Table 6:** Colorimetric analysis of (co-)microencapsulated powders.

Variants	L*	a*	b*	c*	h*
WPI-MD	46.29 ± 0.15 ^a^	19.20 ± 0.13 ^b^	6.57 ± 0.01 **^b^**	20.29 ± 0.12 ^b^	0.33 ± 0.00 ^a^
WPI-MD-LC	44.09 ± 0.25 ^b^	25.01 ± 0.65 ^a^	6.89 ± 0.11 ^a^	25.94 ± 0.66 ^a^	0.27 ± 0.00 ^b^

WPI-MD—microencapsulated extract from cornelian cherry fruits with whey protein isolates and maltodextrin; WPI-MD-LC—co-microencapsulated extract from cornelian cherry fruits with whey protein isolates, maltodextrin, and *L. casei*. For each color parameter (L*, a*, b*, c*, h*), values in the same column that do not share the same lowercase letters (^a^, ^b^) are statistically different at *p* < 0.05, based on the Tukey test.

**Table 7:** Storage stability of biologically active compounds of microencapsulated powders.

Variants	Storage Time (Days)	TAA (mMol/g D.M)	TPC (mg GAE/g D.M.)	TFC (mg CE/g D.M.)	TAC (mg C3G/g D.M.)
WPI-MD	7	59.33 ± 0.29 ^a,A^	10.10 ± 0.05 ^a,B^	4.97 ± 0.39 ^a,A^	10.88 ± 0.39 ^a,A^
	14	55.92 ± 0.92 ^b,A^	9.64 ± 0.14 ^b,B^	4.95 ± 0.29 ^a,A^	10.83 ± 0.65 ^a,B^
	21	49.51 ± 1.36 ^c,A^	9.16 ± 0.11 ^c,B^	4.94 ± 0.06 ^a,A^	10.64 ± 0.06 ^a,B^
	28	31.81 ± 0.41 ^d,A^	8.28 ± 0.04 ^d,B^	4.86 ± 0.25 ^a,A^	10.15 ± 0.12 ^a,B^
WPI-MD-LC	7	59.49 ± 0.25 ^a,A^	12.70 ± 0.08 ^a,A^	5.08 ± 0.13 ^a,A^	12.23 ± 0.80 ^a,A^
	14	56.14 ± 0.26 ^b,A^	12.62 ± 0.21 ^a,A^	5.13 ± 0.08 ^a,A^	12.21 ± 0.21 ^a,A^
	21	49.07 ± 0.15 ^c,A^	12.56 ± 0.11 ^a,A^	5.11 ± 0.13 ^a,A^	12.18 ± 0.93 ^a,A^
	28	32.69 ± 0.80 ^d,A^	12.49 ± 0.03 ^a,A^	4.96 ± 0.15 ^a,A^	12.13 ± 0.55 ^a,A^

WPI-MD—microencapsulated extract from cornelian cherry fruits with whey protein isolates and maltodextrin; WPI-MD-LC—co-microencapsulated extract from cornelian cherry fruits with whey protein isolates, maltodextrin, and *L. casei*. Different lowercase letters (^a^, ^b^, ^c^, ^d^) indicate significant differences (*p* < 0.05) for each phytochemical analysis (TAA, TPC, TFC, and TAC) of WPI-MD and WPI-MD-LC powders during storage (days), while uppercase letters (^A^, ^B^) indicate significant differences (*p* < 0.05) between powder variants at the same storage time (day) for each phytochemical analysis, based on ANOVA results followed by the Tukey test.

## Data Availability

Dataset available on request from the authors.
